# Aptamer Technology: Adjunct Therapy for Malaria

**DOI:** 10.3390/biomedicines5010001

**Published:** 2017-01-04

**Authors:** Nik Abdul Aziz Nik Kamarudin, Nurul Adila Mohammed, Khairul Mohd Fadzli Mustaffa

**Affiliations:** Institute for Research in Molecular Medicine (INFORMM), Universiti Sains Malaysia, Health Campus, Kubang Kerian, 16150 Kelantan, Malaysia; nkabdaziz@gmail.com (N.A.A.N.K.); diela.1123@gmail.com (N.A.M.)

**Keywords:** *Plasmodium falciparum*, malaria, cytoadherence, adjunct therapy, aptamer

## Abstract

Malaria is a life-threatening parasitic infection occurring in the endemic areas, primarily in children under the age of five, pregnant women, and patients with human immunodeficiency virus and acquired immunodeficiency syndrome (HIV)/(AIDS) as well as non-immune individuals. The cytoadherence of infected erythrocytes (IEs) to the host endothelial surface receptor is a known factor that contributes to the increased prevalence of severe malaria cases due to the accumulation of IEs, mainly in the brain and other vital organs. Therefore, further study is needed to discover a new potential anti-adhesive drug to treat severe malaria thus reducing its mortality rate. In this review, we discuss how the aptamer technology could be applied in the development of a new adjunct therapy for current malaria treatment.

## 1. Introduction

Malaria is a life-threatening disease caused by parasites transmitted to humans through the bite of infected *Anopheles* mosquitoes. The disease mostly affects children, pregnant women, non-immune persons, and individuals with chronic diseases such as human immunodeficiency virus and acquired immunodeficiency syndrome (HIV/AIDS). Malaria causes complications such as severe anemia, metabolic acidosis, and cerebral malaria, often leading to death if not treated within 24 h [1,2].

The World Health Organization (WHO) reported there were 438,000 deaths caused by malaria worldwide, especially in the endemic areas such as Africa [3]. In humans, malaria is caused by five distinct *Plasmodium* species, namely *P. falciparum*, *P. vivax*, *P. malariae*, *P. knowlesi*, and two sub-species of *Plasmodium ovale* (*P. o. curtisi* and *P. o. wallikeri*) [4,5,6]. Of these, *P. falciparum* causes the most severe disease due to higher parasitemia, and it is responsible for the massive burden of global mortality and morbidity [7,8]. Despite extensive interventions by WHO to prevent, control and eliminate malaria, the transmission of the disease continues in many countries around the world. The interventions consist of an array of drugs, insecticides, diagnostics, and understanding of the breeding site criteria [9]. Other factors that contribute to the prevalence of malaria include increased transmission risks among people who are non-immune to the disease, the growth in international travel and migration, and the escalation of drug-resistant parasites [10]. However, the underlying mechanism that contributes to malaria severity in a patient is still not well understood, adding to the difficulty in curbing the disease’s progression.

Several drugs are available for malaria treatment including chloroquine, sulfadoxine/pyrimethamine (SP), and quinine, which are working well in many parts of the world. Unfortunately, there is a grave concern that the malaria parasites have developed a widespread resistance to anti-malarial drugs, especially in the endemic regions [11,12]. Anti-malarial drug resistance has been observed for *P. falciparum*, *P. vivax* and *P. malariae* in most parts of the world [13]. The SP resistance is seen in Papua New Guinea, Thailand, Indonesia, Madagascar, Iran, Afghanistan, India, and Pakistan whereas chloroquine resistance is observed in Southeast Asia and South America, including Africa and India [14,15]. Though the artemisinin-based combination therapies (ACTs) were used to treat malaria globally, the artemisinin-resistant parasite was detected when treatment was given to symptomatic malaria patients, and clearance of the parasite from the bloodstream was delayed [16]. The artemisinin-resistant parasite was first discovered in the province of Pailin and these days it can be found in several other countries such as Cambodia, Myanmar, Thailand, and Vietnam. Therefore, the understanding of the disease epidemiology and genetics of malaria are crucial in order to control the spread of parasite resistance to anti-malaria drugs [17,18,19,20].

As the threat of anti-malarial drug resistance grows, there is increasing pressure to develop alternative treatments. Therefore, taking into consideration the ability of the parasites to infect erythrocytes, the development of an anti-adhesive drug as an adjunct therapy to treat severe malaria could be considered. In this review, we shall focus on how the aptamer technology can be explored as a potential anti-adhesive therapy for malaria.

## 2. Pathogenicity of Malaria

Malarial parasites have a complex life cycle involving sexual and asexual reproductive stages. The sexual stage takes place inside the mosquito vector. An infected female *Anopheles* mosquito injects a sporozoite of *P. falciparum* into the human host, which invades the host’s hepatocytes. The asexual life cycle of the parasite begins within 8 to 10 days, when merozoites are formed. The merozoites are then released into the bloodstream and rapidly invade normal erythrocytes. During the asexual blood stage, the merozoites develop into the ring-stage, the pigmented-trophozoite stage, and the schizont-stage inside the infected erythrocytes (IEs) within 48 h. The cycle is repeated through the replication and the release of new merozoites to invade other uninfected erythrocytes. Repeated cycles of IEs invasion, replication, and merozoite release result in an exponential growth of the parasite leading to the progression of the disease.

**Figure 1:**
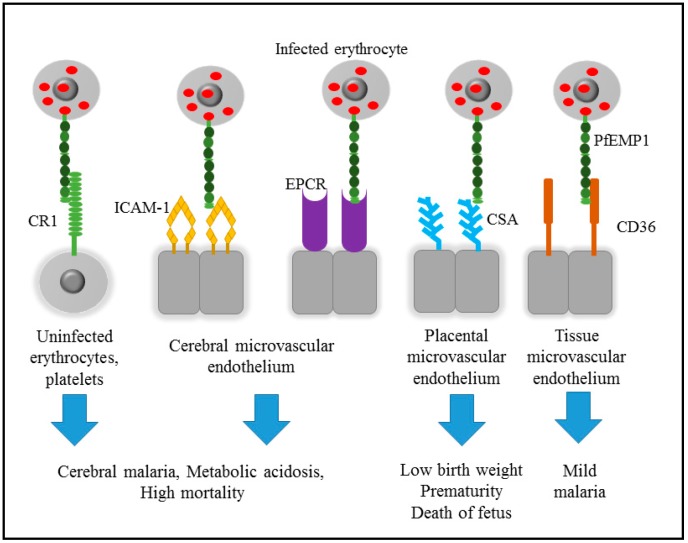
The effect of infected erythrocytes sequestration by various endothelial surface receptors and their disease phenotype. The diagram is modified from Penman et al. (2008) and Cabrera et al. (2014) [26,27].

Most of the symptoms and clinical complications of malaria are observed during the asexual stage. It starts with a high fever and is associated with “flu-like” symptoms followed by a headache, chills and vomiting [21]. However, these symptoms may be mild and difficult to recognize as malaria, even if the falciparum parasites are detected in the blood, especially in uncomplicated malaria [22]. During the asexual stage, the IEs containing pigmented-trophozoite and schizont express a protein-derived adhesion molecule on the surface, *P. falciparum* erythrocyte membrane protein 1 (PfEMP1). The transported protein on the surface of the red blood cell is known to play an important role in the adhesion mechanism to host endothelial tissues and stimulate immune recognition [23,24,25]. Unfortunately, sequestration of the IE to the microvascular surface protein will cause obstruction to the vessel that would subsequently contribute to the development of severe malaria. WHO has defined severe malaria as an abnormal metabolic process, especially in the blood system that creates an acidic condition, hypoxia, and cell necrosis and apoptosis that could be lethal to the infected patient [21]. Figure 1 shows the effect of IE sequestration to various surface receptors such as the complement receptor 1 (CR1), intercellular adhesion molecule 1 (ICAM-1), chondroitin sulfate A (CSA), heparan sulphate, cluster of differentiation 36 (CD36), and endothelial protein C receptor (EPCR), which determine the disease outcomes.

## 3. Cytoadherence of Infected Erythrocyte

**Figure 2:**
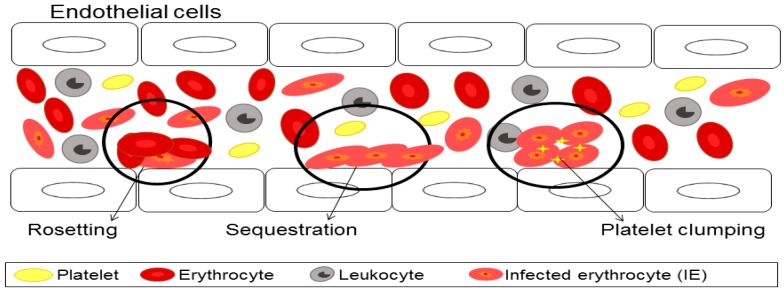
The cytoadherence of infected erythrocytes to red blood cell and endothelial cell promotes the formation of rosetting, sequestration and platelet clumping. These infected erythrocytes (IEs) bind to a number of host receptors on the endothelial cell through tethering, rolling, and adhesion of IE on the surface. This cytoadherence of IEs to the host endothelial surface receptor prevents parasite clearance by the spleen. The diagram is modified from Rowe et al. (2009) [21].

Cytoadherence is believed to be one of the factors that cause severe malaria. In cytoadherence, IEs acquire novel adhesive properties which are sequestration (interaction of IEs with endothelial receptor ligands), rosseting (interaction of IEs with uninfected erythrocytes), and platelet clumping (interaction of IEs with platelets causing them to bind to other IEs) as shown in Figure 2 [28]. PfEMP1 is involved in the cytoadherence process which enables the IEs to bind to a number of host receptors on the endothelial cell through tethering, rolling, and adhesion of IE on the surface [21,29,30]. The cytoadherence of IEs to the host endothelial surface receptor prevents parasite clearance by the spleen [21,31].

CD36 is known as a multiligand scavenger receptor that mediates the binding and uptake of a wide variety of particulate ligands such as oxidized low-density lipoproteins, bacteria, β-amyloid plaque, and apoptotic cells by macrophages [32,33]. In malaria, cytoadherence mediated by CD36 may contribute to the dysfunction of certain organs such as lung, liver, and kidney by impairing the microcirculatory blood flow [34,35,36]. Interestingly, this protein is expressed at low level in the brain where it is not inducible by inflammatory cytokines and also indirectly supports the cytoadhesion of the IEs to the microvascular brain, therefore preventing severe malaria [28,37].

In cerebral malaria (CM), it has been shown that there is an association between IE binding to ICAM-1, although there is no explanation on how the mechanism of IEs cytoadherence in the brain leads to CM [28,38]. A recent study has successfully identified the conserved domain cassette (DC) structure, DC4, that plays a role in PfEMP1 binding to ICAM-1 by the duffy-binding–like (DBL), DBLβ3_D4 (domain 4). This DC4 has also been shown to be linked to the pathogenesis of the severe form of the disease [39,40]. A study by Berger et al. showed that DC5-containing PfEMP1 bound to platelet and endothelial cell adhesion molecule 1 (PECAM1) has been associated with CM [41,42]. However, the binding of IEs to PECAM1 was also found in uncomplicated malaria patients, where DC5-containing PfEMP1 was also expressed by the parasite when not selected for adhesion to brain endothelial tissue [43,44,45].

Several studies have demonstrated that endothelial dysfunction and resulting inflammation preferentially affected the brain, but the mechanism behind it is still unclear [28]. Post-mortem studies conducted by several groups have shown a small haemorrhage in the brain which was associated with the platelets and thrombin or fibrin, but their role in the coagulation and specificity in CM is still being debated [46,47,48]. The endothelial protein C receptor (EPCR) and thrombomodulin (TM) play a major role in regulating the coagulation, inflammation, endothelial barrier function, and neoprotection in the anticoagulant protein C pathway [49,50,51]. These proteins are expressed at low level in the brain compared to other organs [52,53].

The association between the loss of local EPCR and TM with CM are caused by the sequestration of IEs, which disturbs the coagulation and inflammatory mechanism [48]. In an excessive clotting cascade, the thrombomodulin is bound to the thrombin and forms a thrombomodulin-thrombin complex. This complex then activates protein C to form activated protein C (APC) and this process is strongly accelerated by EPCR [54,55]. The APC can be exerted as anticoagulant, anti-inflammatory, antiapoptotic, and vasculoprotective signal through the protease activated receptor 1 (PAR1). In CM, the protein C pathway becomes impaired causing the level of thrombin to increase and eventually distrupt the endothelial barrier function, increasing the inflammation and coagulation signals.

Furthermore, Lavsteen et al. recently revealed that PfEMP1 domain cassettes 8 and 13 can bind to EPCR endothelial receptor near or at the same region as APC, which can inhibit APC-mediated EPCR-dependent cytoprotective effects on endothelial cells [43,45,56]. The authors also demonstrated that the EPCR displayed a stronger binding in children with severe malaria compared to children who have uncomplicated malaria.

## 4. Introduction to Aptamer Technology

Since 1990, many studies have been conducted using the RNA molecules that specifically bind to many targets, including small molecules, proteins, enzymes, nucleic acids, protein receptors, viruses, and considerably more targets [57]. Single-stranded nucleic acid (ssDNA or RNA) can be folded to form a complex secondary and tertiary structure which can bind to different compounds, molecules and proteins [58]. The term aptamer is derived from Latin and Greek words which are “Aptos”—to fit, and “meros”—part of the region [59]. Aptamers bind to their target via an “induced fit” mechanism, which allows them to tightly bind to the target at high specificity and affinity, similar to antibodies (they’re also known as chemical antibodies) [60,61,62]. Interestingly, aptamers bind to their target through hydrogen bonding, electrostatic interactions, van der Waals forces and shape complement; similar to antibody-antigen recognition and complex formation [63]. Even though both DNA aptamers and RNA aptamers can form complex structures, the RNA aptamer can form more diverse three dimensional (3D) structures compared to DNA aptamers, as RNA contain 2′-OH (hydroxide) group on their ribose sugar [61,64,65].

SELEX (Systematic Evolution of Ligand by Exponential Enrichment) is a method used to isolate aptamers that specifically bind to a target with high affinity through a repetitive amplification and selection process. Previous studies revealed that the SELEX method has been used extensively to isolate high affinity ligands that bind at picomolar and low nanomolar affinities to a wide variety of proteins and cell surface epitopes [66]. For instance, SELEX has been used to isolate cluster of differentiation 4 (CD4), L-selectin, Vascular Endothelial Growth Factor (VEGF), Epidermal Growth Factor Receptor (EGFR) as well as complex targets such as the red blood cell membrane, the membrane-bound nicotinic acetylcholine receptor, and whole virus particles [66,67,68].

In the SELEX technology, there are several crucial steps which must be followed until the aptamer with high specificity and strong affinity to a target can be isolated. (i) The SELEX process starts with a chemical synthesis of a randomized oligonucleotide containing 10^14^ to 10^16^ unique randomized sequences flanked by primer binding regions [56,66]. Usually in SELEX, the length of RNA or ssDNA is within the range of 56 to 120 base pairs depending on how complex the starting nucleic acid pool needs to be [67]. For DNA aptamer selection, the dsDNA should be separated to obtain ssDNA by using several methods such as asymmetric PCR, biotin-streptavidin separation, lamba exonuclease digestion and size-based separation by denaturing polyacrylamide gel electrophoresis (PAGE) [69,70,71]. However, an additional method is needed to synthesize the RNA pool from the DNA pool through in vitro transcription for RNA aptamer selection, as commercialized production is expensive compared to in-house production. The sense primer should be included with the T7 RNA polymerase promoter to allow in vitro transcription to occur [68,69,70].

**Figure 3:**
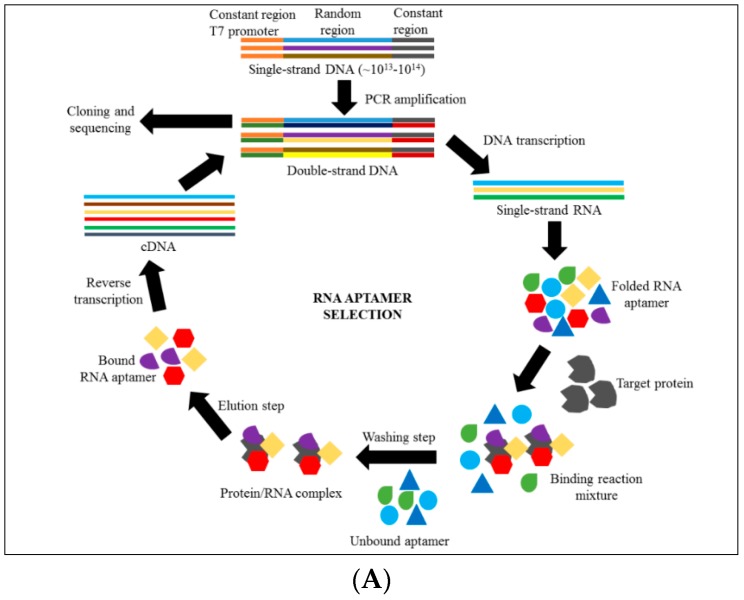

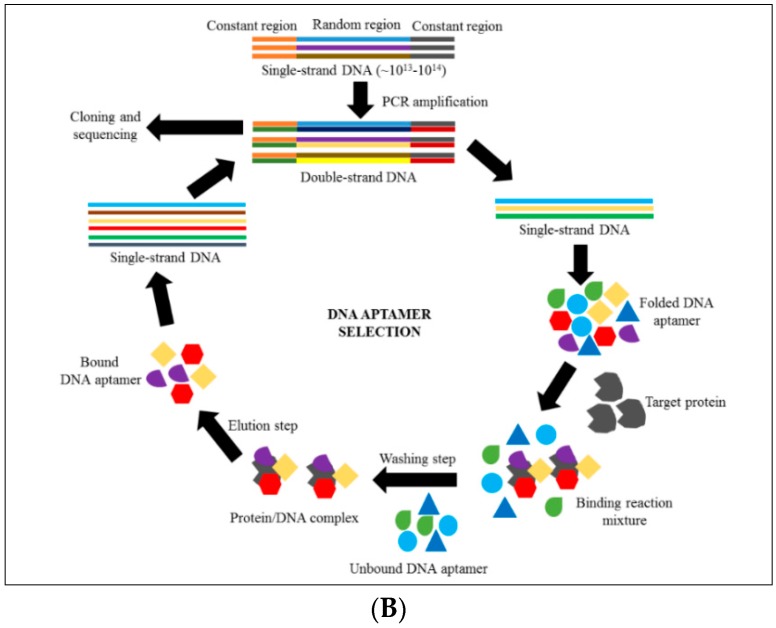
RNA aptamer and DNA aptamer development. (**A**) Isolation of RNA aptamer targeting selected molecules requires an additional step to produce an RNA strand from single-stranded DNA through DNA transcription. Reverse transcription is needed to produce single-stranded DNA to produce an RNA pool for the subsequent SELEX cycle; (**B**) A separation step is required to produce single-stranded DNA from double-stranded DNA prior to the DNA aptamer selection. Commonly, the pool of aptamers begins with 10^13^ to 10^14^ random sequences which are then enriched into several groups of aptamers that specifically to their target. This can be achieved by sequencing the final bound product of SELEX and aligning the sequences. Further study is needed to measure the binding affinity of enriched aptamers to the target molecules.

After that, (ii) the oligonucleotide library is incubated with the target molecules to allow the binding to occur. The selection incubation is commonly performed at room temperature or on ice (4 °C). Other conditions depend on the target aptamer assay development. After the incubation, (iii) the aptamer that bound to the target is separated from unbound aptamer. Plentiful methods have been established, from simple methods (e.g., nitrocellulose filter, affinity chromatography column, microtitre plate-based method) to using high-technology instruments (e.g., Surface Plasmon Resonance (SPR), Capillary Electrophoresis (CE) and flow cytometry) to partition the unbound aptamer from target-bound aptamer [72,73,74,75]. The development of new high-technology instruments has helped scientists to do a SELEX process in only a few hours, instead of over several weeks [76]. The eluted bound aptamers are amplified through a polymerase chain reaction (PCR) and are used for another round of the SELEX cycle. However, for the RNA aptamer, the collected RNA pool should be reverse transcribed before amplification using PCR. The enrichment of the SELEX cycle can be monitored (iv) by adding a fluorochrome reporter at the 5′-sense primer sequence for a DNA aptamer. An RNA aptamer can be tagged either at the 3′-end or the 5′-end, which is an alternative way for radioactive labelling [77,78]. Figure 3 shows the comparison of methods between RNA aptamer and DNA aptamer development. (v) After several iterative rounds of SELEX, the final products of target-bound aptamer are cloned and subjected to sequencing. The sequences are aligned and clustered based on their similarities of homologous sequences and common motif structures. Further investigation is needed to identify the most prominent clusters that bind to a target as potential aptamer candidates.

## 5. Aptamers as Therapeutic Agents Compared with Antibodies

Most of the current therapeutic and diagnostic applications use a monoclonal or polyclonal antibody against a specific antigen or protein. Since aptamer technology was discovered in the early 1990s, numerous studies have been conducted to isolate specific aptamers to specific molecules, especially intercellular molecules, which are unreachable by an antibody. Aptamers have more advantages than antibodies in terms of production, stability, binding affinity, small size, and less immunogenicity [79,80].

Production of monoclonal antibodies (mAb) normally uses a vertebrate organism such as a mouse, goat, or rabbit, and injecting the animals with a specific antigen to produce a specific antibody. Aptamer production does not require organisms for oligonucleotide synthesis [81]. Instead, instruments that are commonly used in the molecular laboratory such as a thermal cycler are utilised. Therefore, oligonucleotide synthesis offers a huge benefit in manipulating the process of direct evolution through in vitro selection of the aptamer for the target molecules.

Moreover, the in vitro production of monoclonal antibodies (mAb) using mammalian cell culture is laborious and very expensive. The process involves a large number of colonies and requires confirmation of antibody activity by immunoassay in each new batch produced, as the same antibodies tend to change in different batches [62,79,82]. Since the aptamer is chemically synthesized, it can be synthesized with greater accuracy and reproducibility with a lower variation [83]. Aptamer development companie such as Archemix’s Stanton have shown that the manufacture of custom aptamers will cost $50 or less per gram on manufacturing scale [84]. Custom aptamer manufacturing can be completed within two days as compared to months for human monoclonal antibody production at an average cost $300/g [84,85].

As mentioned earlier, an aptamer can reach target molecules which are difficult to reach by antibodies such as those inside tissues or intercellular molecules due to their size (8–25 kDa) compared to the antibody (approximately 250 kDa). The intercellular molecules allow the aptamer to penetrate the tissue more efficiently to reach their target in vivo in comparison to the larger size of the protein antibody.

Since aptamers are a single stranded nucleic acid, theoretically they should not be recognised by the human immune system as foreign molecules. However, a study by Eyetech Study Group showed a slight immunogenicity of a VEGF-specific aptamer when given to a monkey at a 1000-fold higher dose than the therapeutic dose [86]. This is comparable to antibodies which are significantly immunogenic, especially following repeated dosing [87].

Furthermore, the antibody is well known for its irreversible denaturation that can cause it to lose its binding ability to a specific target, as its tertiary structure is lost when exposed to high temperatures. Aptamers are more thermally stable even after 95 °C denaturation [79,88]. Thus, aptamers are able to recover their native structure over repeated cycles of denaturation and renaturation that allow them to be used under a wide range of assay conditions [83].

A limitation of aptamers compared to antibodies is that the RNA aptamers are susceptible to nuclease degradation at the 2′-OH on the ribose sugar of nucleic acid. Nevertheless, it still can be improved through chemical modification by substitution of the 2′-OH group with 2′-fluoro pyrimidine, 2′-amino pyrimidine, or 2′-*O*-methoxy pyrimidine and purine. This modification not only increases nuclease resistance but also allows stronger aptamer binding with the target and increased binding specificity [89].

## 6. Discovering Anti-Cytoadhrence through Aptamer Technology

In terms of aptamer generation for anti-adherence of the malaria parasite to the microvascular endothelial surface receptor, the target should be identified. In other words, it is crucial to determine whether a purified recombinant protein or a whole cell surface should be used. Similar to protein-based antibody development, the recombinant protein of a selected surface receptor such as ICAM-1, CD36, and VCAM-1 can be expressed in the prokaryotic or eukaryotic system by tagging them with certain affinity molecules such as histidine, immunoglobulin, glutathione-*S*-transferase, and streptavidin for protein purification and surface immobilization purposes [39,90,91]. However, often a highly glycosylated protein, purified protein or peptide cannot be folded into the correct 3D structures in their native conditions due to post-translational modification [92]. The newly synthesized aptamer may be incapable of recognizing the selected target which would result in the failure of selection. Therefore, a modified SELEX technology using whole living cells or Cell-based SELEX (Cell-SELEX) could be used to increase the chances of success [93].

However, a previous study revealed that Cell-SELEX caused a low aptamer enrichment efficiency because a number of proteins or molecules co-expressed on the cells [61]. This might cause the selectivity of the aptamer to the target to become less specific, and cause a low affinity to the target. To overcome this problem, another method of SELEX can be used by combining the Cell-SELEX method with a protein-based SELEX known as Cross-Over SELEX or Hybrid-SELEX. This Hybrid-SELEX method has been used to isolate an RNA aptamer specific to Tenascin-C and a DNA aptamer specific to CD30-expressing lymphoma tumour cells, respectively [94,95]. When using the Cross-Over SELEX, the aptamer pool is initially incubated with a cell-expressing selected protein or molecule, and then the bound aptamer is collected. After amplification, the subsequent aptamer is incubated with a purified protein or molecule of interest. This approach allows for higher selectivity of the aptamer in recognizing and binding to its target protein.

Therefore, a potential anti-cytoadherence could be developed using Cross-Over SELEX by combining the purified recombinant protein (e.g., ICAM-1 protein) and human umbilical vein endothelial cells (HUVEC). HUVEC is a cell line that expresses small levels of ICAM-1 but is CD36-deficent. However, tumour necrosis factor (TNF) will promote the up-regulation of expression of ICAM-1 [96,97]. After several iterative rounds of selection against recombinant ICAM-1 protein and HUVEC, the isolated aptamer is then further investigated for its ability to inhibit and reverse the binding of IEs to ICAM-1 using static or flow assay.

Study on inhibiting the IEs binding property to isolated aptamers can be performed by incubating the mature stage of Plasmodium IEs culture with immobilized recombinant ICAM-1 protein or HUVEC which previously blocked the isolated aptamer. After incubating and washing steps, the remaining parasite will be calculated and compared with control (untreated with aptamer). Aptamer that shows significant reduction in the number of bound IEs might possess the cytoadherence inhibition property. Study on reversing the IEs binding property can be performed by introducing the aptamer candidate into the assay containing IEs culture bound to immobilized protein or cells. Any significant reduction in the number of bound IEs might indicate that the aptamer has the ability to reverse the IEs binding to the receptor protein.

The potential aptamer that is able to inhibit or reverse the binding of IEs from endothelial protein receptor can allow the clearance by the spleen and relieve microvascular obstruction in severe malaria patients [21]. This adjunct therapy might have a clinical benefit to reduce mortality rates for severe malaria even after giving anti-malaria drugs [98].

## 7. Current Aptamer Development for Malaria Therapy

Even though the mechanism on how parasite cytoadherence to microvasculature can result in the severity of the malaria disease is yet to be comprehended, post-mortem studies have shown that the presence of IE in the microvessels is associated with the disease’s pathology [22]. The cytoadherence and rosetting of IE to endothelial surface receptors such as ICAM-1, CD36, heparin and chondroitin sulfate A by PfEMP1 could be used to innovate a new therapy that has the capability to block or reverse the binding of IEs. By identifying the conserved region of PfEMP1 which is responsible for rosette formation, Barfod et al. successfully isolated the specific RNA aptamer targeting DBLα after eight rounds of selection starting with 5 × 10^14^ unique sequences [99]. They have shown that the isolated RNA aptamer reduced rosette formation by 35% at 33 nM concentration and by 100% at 387 nM concentration during an in vitro study using a high rosette-forming strain, *P. falciparum* FCR3S1.2 [99,100].

Current treatment of malaria involves drug usage such as chloroquine, quinine, sulfadoxine/pyrimethamine, and artemisinin to disturb the biological mechanism of the live parasite in the human blood cell from surviving due to the heme toxicity. The drugs prevent the heme from crystallizing to hemozoin through the formation of deprotonated drug, which caps the hemozoin to prevent further crystallization of the heme. This heme molecule is highly toxic to the cell and leads to the dysfunction of the membrane resulting in cell lysis and parasite cell autodigestion. Therefore, to avoid this toxic molecule, the heme molecules (Fe^2+^) oxidize to form hemozoin (Fe^3+^) which precipitates the inside of the parasite vacuoles, also known as a malaria pigment [101]. Using the hemoglobin degradation mechanism, Okazawa et al. successfully isolated the DNA aptamer that specifically binds to the heme molecules to interfere with the heme-detoxification in *P. falciparum* [102,103]. The DNA aptamer selection was carried out at pH 7–8. Fortunately, the selected heme-binding DNA aptamers were able to bind similarly to the heme molecules at an acidic pH, where the parasite growth was significantly reduced after 72 h in the culture when compared to the non-heme binding DNA aptamer [104]. This finding showed promise for the isolated DNA aptamer’s probability of being used as a therapeutic tool to improve the antimalarial drugs. Table 1 shows the summary of previously developed aptamers for malaria treatment targeting parasite expressed protein (DBLα) and heme molecules.

## 8. Conclusions

In the South East Asian Quinine Artesunate Malaria Trial (SEQUAMAT) study, Dondrop and colleagues revealed that there is correlation between high parasite burdens in the blood vessel and the severity of malaria where 15% of patients died even after they were given an artesunate treatment [105]. The study indicated that there is an accumulation of IEs in the tissues which were linked to the disease’s severity [105]. This proved that *P. falciparum* cytoadherence has a key role in the pathogenesis of life-threatening malaria and it could be targeted by a drug or a small molecule therapy.

Several mAb were discovered to be capable of blocking or inhibiting IE cytoadherence such as mAb OKM5 which can bind to an epitope of the ectodomain region of CD36 to block the cytoadherence of IEs to CD36 [106]. Meanwhile, mAb 15.2 against the L42 loop of domain 1 of ICAM-1 has been found to be able to inhibit the cytoadherence of IEs to ICAM-1 [107]. However, mAb also possess some limitations such as high immunogenicity, increased cost of production, large in size, thermally unstable and difficult to chemically modify [108].

Therefore, discovering a new technology such as aptamer development is vital to inhibit and reverse parasite binding on endothelial cells. Current aptamer development for malaria targets PfEMP1 which is associated with rosette formation and heme metabolism. Unfortunately, an aptamer targeting the host endothelial surface receptors to block or reverse the binding of IEs is yet to be explored. In this review, a number of endothelial receptors were mentioned and could be potentially used for future studies to isolate the aptamer to select the receptor protein, a potential for the malaria anti-adherence.

## Figures and Tables

**Table 1:** Summary of previously developed aptamers for malaria treatment.

Aptamer	Target	Function	Result	Reference
RNA	Surface protein of PfEMP1—DBLα domain	To disrupt the rosette formation between infected erythrocyte with normal erythrocyte.	Isolated RNA aptamer reduced rosette formation by 35% at 33 nM concentration and 100% reduction at 387 nM concentration during in vitro study using high rosette-forming strain, *P. falciparum* FCR3S1.2	[99]
DNA	Heme group	To interfere with heme-detoxification and growth of *P. falciparum* in infected erythrocyte.	Parasite growth was significantly reduced after 72 h in a culture when compared with the control	[102]
